# Porcine *FRZB* (sFRP3) Negatively Regulates Myogenesis via the Wnt Signaling Pathway

**DOI:** 10.3390/ani16020276

**Published:** 2026-01-16

**Authors:** Jingru Nie, Yu Fu, Xin Hao, Dawei Yan, Bo Zhang, Hao Zhang

**Affiliations:** 1 Frontiers Science Center for Molecular Design Breeding (MOE), China Agricultural University, Beijing 100193, China; 13627606200@163.com; 2 National Tropical Palm Germplasm Reserve/International Joint Research Center for Date Palm Resource Evaluation and Cultivar Propagation, Coconut Research Institute of Chinese Academy of Tropical Agricultural Sciences, Wenchang 571339, China; fuyu6486@163.com (Y.F.); haoxin0331@163.com (X.H.); 3 National Key Laboratory for Tropical Crop Breeding, Sanya Research Institute of Chinese Academy of Tropical Agricultural Sciences, Sanya 572025, China; 4 College of Animal Science and Technology, Yunnan Agricultural University, Kunming 650201, China; 1995027@ynau.edu.cn

**Keywords:** C2C12, *FRZB*, muscle development, myogenesis, pig, sFRP3, Wnt signaling

## Abstract

Secreted frizzled-related protein 3 (sFRP3/*FRZB*) is a soluble Wnt antagonist with established roles in skeletal development, however, its specific function in myogenesis remains underexplored. This study investigated the regulatory role of *FRZB* in muscle development, hypothesizing that it contributes to breed-specific growth differences in pigs. We examined *FRZB* expression in fetal tissues of slow-growing (Tibetan and Wujin) and fast-growing (Large White) pigs, and assessed its function in C2C12 myoblasts via siRNA-mediated knockdown. *FRZB* was widely expressed across porcine fetal tissues, with significantly higher abundance in the longissimus dorsi of slow-growing breeds. In vitro, *FRZB* silencing significantly enhanced myoblast proliferation and migration. Furthermore, knockdown accelerated differentiation and promoted the formation of longer, thicker multinucleated myotubes, accompanied by the upregulation of myogenic (*MyoD*, *MyoG*, *MyHC*) and fusion (*β1-integrin*, *Myomaker*) markers. Transcriptional profiling revealed a shift toward hypertrophy (*Fst* and *Nog* upregulation) and away from atrophy (*Atrogin1* downregulation). These findings identify *FRZB* as a negative regulator of myogenesis via the Wnt signaling pathway. The elevated expression in indigenous breeds suggests *FRZB* may impose a molecular constraint on muscle development, highlighting its potential as a candidate gene for regulating carcass traits.

## Simple Summary

Skeletal muscle growth is the primary factor determining meat production in the pig industry. A gene called *FRZB* is known to affect bone development, but its specific role in muscle formation is not well understood. This study aimed to investigate how *FRZB* influences muscle growth and whether it contributes to the size differences between pig breeds. We compared slow-growing indigenous pigs with fast-growing commercial pigs and found that the slow-growing breeds had significantly higher levels of *FRZB* in their loin muscles during development. To understand its function, we reduced the levels of *FRZB* in muscle cells in the laboratory. We observed that cells with lower *FRZB* levels multiplied faster and formed larger, thicker muscle fibers. Our results suggest that *FRZB* acts like a natural “brake” on muscle growth by blocking specific growth signals. This research is valuable to society because it identifies a biological factor that limits meat yield. Understanding this mechanism could help animal breeders select pigs with better muscle growth traits, ultimately improving efficiency in meat production.

## 1. Introduction

Pork is the most essential meat source in China, and modern swine breeding increasingly prioritizes the balance between growth performance and superior meat quality. Since skeletal muscle growth and fiber characteristics are primary factors determining meat production and quality, identifying key genetic regulators of myogenesis is essential for improving carcass traits across diverse pig breeds [1]. During vertebrate embryogenesis, skeletal muscles in the limbs arise from muscle progenitor cells derived from somites [2]. Postnatal muscle growth primarily depends on increases in muscle fiber volume. Muscle development involves a series of biological processes, including myoblast proliferation, differentiation, and fusion, which lead to the formation of multinucleated myotubes that eventually mature into functional muscle fibers [3]. The growth of these fibers is tightly regulated by specific muscle-related transcription factors [4] and protein kinases [5]. Among them, myogenic regulatory factors (MRFs) serve as central nodes within the transcriptional network and directly drive skeletal muscle development [6]. Cyclin-dependent kinases further regulate the myoblast cell cycle, balancing proliferation with differentiation [7,8]. The temporal and spatial expression of these regulators is strictly controlled to ensure the orderly progression of myogenesis [1,9].

Our previous study revealed that the expression level of the frizzled-related protein (*FRZB*) gene in the longissimus dorsi (LD) muscle of slow-growing Chinese indigenous pig breeds (Diannan small-eared pig and Tibetan pig, DSP-TP) was approximately 3.8-fold higher than that observed in fast-growing introduced breeds (Landrace and Yorkshire, LL-YY) [10]. *FRZB* encodes secreted frizzled-related protein 3 (sFRP3), which is a member of the sFRP family involved in the Wnt signaling pathway. sFRP3 contains a cysteine-rich domain (CRD) homologous to the Wnt-binding site of frizzled receptors, enabling it to competitively disrupt Wnt-frizzled interactions [11]. Members of the sFRP family are generally recognized as Wnt signaling inhibitors [12]. In China, the physiological dichotomy of the swine industry is best exemplified by the contrast between indigenous breeds and commercial breeds. The TP, native to high altitudes, is an evolutionary marvel adapted to extreme hypoxia, exhibiting slow growth but exceptional pork flavor. The Wujin pig (WJ), a classic “fat-type” breed, is celebrated for its superior intramuscular fat deposition and its role in premium ham production. In contrast, the Large White (LW) pig represents the global standard for rapid muscle hypertrophy and feed efficiency [13,14]. Investigating the molecular basis of these divergent growth phenotypes, particularly the role of *FRZB*, provides a roadmap for balancing production speed with meat quality.

Canonical Wnt/β-catenin signaling promotes the expansion of myogenic progenitors and drives the expression of MRFs during both fetal development and regeneration [15,16,17]. In contrast, increasing the levels of extracellular Wnt antagonists can impede myogenesis. For example, transplacental delivery or misexpression of sFRP3/*FRZB* in embryos reduces myofiber formation in a dose-dependent manner and blocks somitic myogenesis upstream of myogenic differentiation antigen (*MyoD*) [18,19]. These findings suggest that *FRZB* functions as a potential brake in myogenesis. Beyond the muscle, canonical Wnt signaling inhibits adipogenic differentiation, whereas Wnt antagonism promotes adipogenesis [20,21,22]. Therefore, tissue-specific *FRZB* expression levels may influence the balance between muscle growth and fat deposition in pigs.

Recent studies have further highlighted the importance of Wnt signaling in muscle biology. For example, Wnt/β-catenin signaling has been identified as a key mediator in muscle-bone crosstalk, influencing both tissue regeneration and homeostasis [23]. A comprehensive review also highlighted new insights into Wnt signaling pathways in development and disease, emphasizing their interplay with Notch, TGF-β, and Hippo pathways [24]. However, direct functional studies on *FRZB* in porcine muscle development remain limited. In the pig genome, *FRZB* is located on chromosome 15 (*Sscrofa11.1*: ~88.33–88.38 Mb) and is composed of multiple exons. Natural variations in its regulatory regions are associated with growth-related traits in pigs [25].

To bridge this knowledge gap, this study aimed to characterize the expression profile of *FRZB* in fetal tissues of diverse pig breeds and to elucidate its regulatory function in myoblast proliferation and differentiation using a C2C12 model. Given the established role of canonical Wnt signaling in promoting myogenesis, we hypothesized that the differential expression of *FRZB* might act as a critical molecular switch modulating myoblast behavior, thereby contributing to the phenotypic variations in muscle growth observed between pig breeds.

## 2. Materials and Methods

### 2.1. Experimental Materials

All animal procedures followed the National Research Council Guide for the Care and Use of Laboratory Animals and were approved by the Institutional Animal Care and Use Committee (approval number: AW80203202-1-1). Tibetan (TP) and Wujin (WJ) pigs, two indigenous Chinese breeds with relatively slow growth rates, and Large White (LW) pigs, a fast-growing commercial breed, were reared at the Tibet Agriculture and Animal Husbandry College. The pregnant sows were raised in semi-closed cement floor pigsties under standardized environmental conditions. The sows were fed twice daily (09:00 and 16:00) with a quantitative allowance based on their gestational stage, while water was provided ad libitum. All animals were immunized and dewormed according to standard farm protocols. The dietary formula was designed based on the Chinese pig feeding standard (NY/T 65-2004) [26] for gestating sows. The detailed formula composition and nutritional levels are listed in Supplementary Table S1. The pregnant sows were slaughtered at 60 days post-insemination, and at least six fetuses (mixed sex) from each breed (TP, WJ, and LW) were collected. Fetuses were selected randomly based on normal morphology and comparable body size. Multiple fetal tissues, namely longissimus dorsi (LD), back fat (BF), kidney, heart, lung, hypothalamus, leg muscle, liver, brain, and intestine (jejunum), were collected for semi-quantitative reverse transcription PCR (sqRT-PCR). Fetal LD tissues (12th rib region) were collected for sqRT-PCR and quantitative real-time PCR (qRT-PCR).

### 2.2. C2C12 Cell Culture and RNA Interference

C2C12 myoblasts (Type Culture Collection of the Chinese Academy of Sciences, China) were cultured in Dulbecco’s modified Eagle’s medium (DMEM; Gibco, Watham, MA, USA) supplemented with 10% fetal bovine serum (FBS; Gibco) and 1% penicillin/streptomycin (Gibco). Cell differentiation was induced by culturing the cells in DMEM supplemented with 2% horse serum (Gibco) and 1% penicillin/streptomycin. To suppress *FRZB* expression, we designed small interfering RNAs (siRNAs) based on the *FRZB* gene sequence and selected the highest-scoring candidates (siRNA-1127, siRNA-1251, and siRNA-1391) from the design platform, with the numerical identifiers of each siRNA corresponding to its position relative to the *FRZB* transcriptional start site. The *FRZB* siRNA sequences were as follows: siRNA-1127, sense: CCGGAACAAUUACAACUAUTT, antisense: AUAGUUGUAAUUGUUCCGGTT; siRNA-1251, sense: GACACCGUCAAUCUUUAUATT, antisense: UAUAAAGAUUGACGGUGUCTT; and siRNA-1391, sense: GGAUCGGCUUGGUAAGAAATT, antisense: UUUCUUACCAAGCCGAUCCTT; si-NC, sense: UUCUCCGAACGUGUCACGUTT, antisense: ACGUGACACGUUCGGAGAATT. Cell transfection was performed using Lipofectamine 2000 (Invitrogen, Waltham, MA, USA) according to the manufacturer’s instructions. For differentiation assays, at 24 h post-transfection, the growth medium was replaced with differentiation medium (DMEM supplemented with 2% horse serum) to induce myogenic differentiation. Based on the knockdown efficiency results (verified by qRT-PCR), siRNA-1127 exhibited the strongest inhibitory effect and was thus selected for all subsequent functional assays.

### 2.3. RNA Extraction and mRNA Level Measurement

Total RNA was extracted from the cells and tissues using TRIzol reagent (Invitrogen). Briefly, TRIzol was added to the samples for ice-bath lysis or homogenization until tissue or cell debris was no longer visible. RNA was then separated from protein and DNA via phenol-chloroform extraction, precipitated with isopropyl alcohol, and collected via centrifugation (4 °C, 12,000 rpm, 15 min). The concentration and purity of RNA were determined using a NanoDrop 2000 spectrophotometer (Thermo Fisher Scientific, Waltham, MA, USA), with an A260/280 ratio between 1.8 and 2.0. RNA integrity was verified by 1% agarose gel electrophoresis. The extracted RNA was reverse-transcribed using the FastQuant Reverse Transcriptase Kit (TIANGEN, Beijing, China). The mRNA expression levels of the target genes were analyzed using semi-quantitative PCR and qRT-PCR with specific primers (Supplementary Table S2). Primers were designed using the NCBI Primer-BLAST tool [27] based on the specific gene sequences. Expression levels were normalized using the 2^−ΔΔCt^ method [28], with *β-actin* serving as the housekeeping reference gene.

### 2.4. Cell Proliferation Measurement

5-Ethynyl-2-deoxyuridine (EdU; RiboBio, Guangzhou, China) and Cell Counting Kit-8 (CCK-8; Solarbio, Beijing, China) assays were conducted following the manufacturers’ protocols. For the EdU assay, cells transfected for 24 h were incubated with 50 µmol/L EdU for 2 h, fixed with 4% paraformaldehyde (PFA), permeabilized with 0.5% Triton X-100, incubated with Apollo reaction buffer, and counterstained with Hoechst to visualize nuclei. Images were acquired using a microscope (ZEISS, Jena, Germany). For the CCK-8 assay, transfected cells were incubated with 100 µL of 10% CCK-8 solution at 37 °C for 1 h, after which absorbance was measured at 450 nm using a microplate reader (Biotek, Winooski, VT, USA).

### 2.5. Transwell Migration Assay

Cell migration was measured using transwell inserts, as previously described [29]. At 24 h post-transfection, myoblasts were trypsinized and counted using a hemocytometer. The cells were then resuspended in serum-free DMEM and added to the upper chamber (200 µL/well), while 500 µL of DMEM supplemented with 10% FBS was added to the lower chamber as a chemoattractant. After incubation for 12 h at 37 °C in 5% CO_2_, cells remaining on the upper surface of the membrane were carefully removed. The migrated cells on the underside were stained with crystal violet (30–60 min), imaged in five randomly selected fields per well using a microscope, and quantified using ImageJ software (version 1.53t; National Institutes of Health, Bethesda, MD, USA). Three independent biological replicates were analyzed per group.

### 2.6. Immunofluorescence (IF)

Myoblasts and muscle tissues were fixed in 4% PFA (20 min) and permeabilized (0.3% Triton X-100 in PBS for 15 min). After blocking (2 h at RT; Beyotime, Haimen, China), the samples were incubated overnight at 4 °C with primary antibodies against the following: MyHC (M4276, 1:200; Sigma-Aldrich, St. Louis, MO, USA), eMyHC (F1.652, 1:50; DSHB, Iowa City, IA, USA), and laminin (L9393, 1:1000; Sigma-Aldrich). Fluorescent secondaries (SA00013-2/-3, 1:400; Proteintech, Rosemont, IL, USA) were added for 1 h at RT in the dark, after which nuclei were counterstained with DAPI (5 min). Images were acquired using a Leica Q500MC system (Leica, Cambridge, UK).

### 2.7. Statistical Analysis

All quantitative data are presented as the mean ± SD. Prior to statistical analysis, the normality of the data distribution was verified using the Shapiro-Wilk test, and the homogeneity of variance was assessed using Levene’s test. Data conforming to these assumptions were analyzed. Between-group comparisons were performed using a two-tailed Student’s *t*-test, while multigroup comparisons (e.g., pig breed expression data) were performed using one-way ANOVA followed by Tukey’s post-hoc test. A minimum of three independent biological replicates were included unless otherwise indicated. Statistical analyses were performed using SPSS (version 22.0; IBM, Armonk, NY, USA). The significance thresholds were set at * *p* < 0.05, ** *p* < 0.01, and *** *p* < 0.001.

## 3. Results

### 3.1. Tissue Distribution and Breed-Specific Expression of FRZB in Pigs

**Figure 1:**
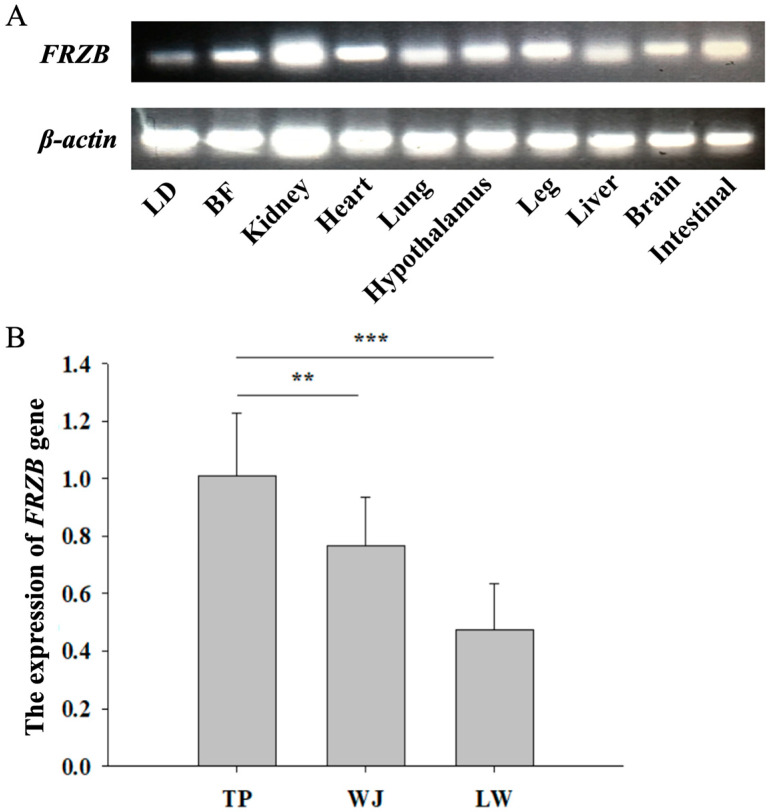
Tissue distribution and breed-specific expression of *FRZB* in pig fetuses. (**A**) Semi-quantitative RT-PCR analysis of *FRZB* expression in fetal tissues of Tibetan pig (TP), showing broad distribution with notable expression in skeletal muscle (longissimus dorsi, LD; back fat, BF). (**B**) Relative *FRZB* expression in LD among TP, Wujin pig (WJ), and Large White (LW) breeds (*n* = 6 per breed). Data are presented as the mean ± SD. Asterisks indicate significant differences (** *p* < 0.01, *** *p* < 0.001) as determined by one-way ANOVA followed by Tukey’s post-hoc test.

*FRZB* expression was broadly detected across multiple fetal tissues of TP, including the LD, BF, kidney, heart, lung, hypothalamus, leg muscle, liver, brain, and intestine (jejunum). Notably, while high expression levels were observed in the kidney and leg muscle, clearly detectable expression was also confirmed in the LD and BF (Figure 1A). Comparative analysis revealed that compared with the fast-growing LW, the slow-growing breeds TP and WJ had significantly higher *FRZB* expression in LD (Figure 1B). These findings are consistent with our previous transcriptomic analysis, validating the breed-specific upregulation of *FRZB* in the skeletal muscle of indigenous pigs [10].

### 3.2. FRZB Knockdown Enhances Proliferation of C2C12 Myoblasts

**Figure 2:**
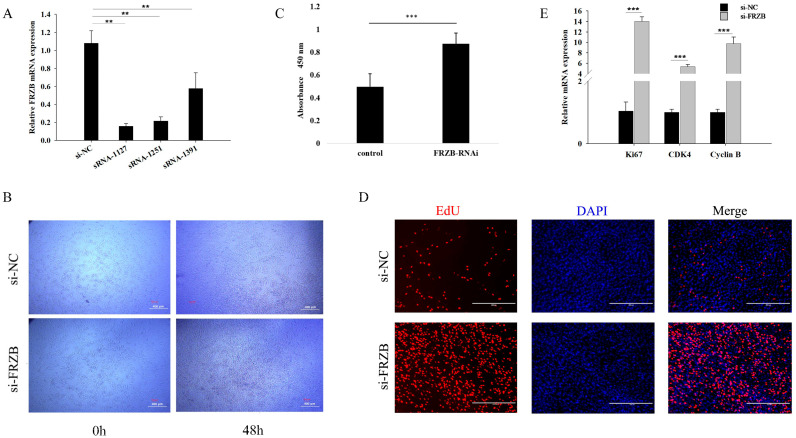
*FRZB* knockdown enhances proliferation of C2C12 myoblasts. (**A**) Knockdown efficiency of different siRNA-*FRZB* fragments measured via qPCR 24 h after transfection. (**B**) Representative phase-contrast images showing cell density in control (si-NC) and *FRZB*-silenced (si-*FRZB*) groups at 0 h and 48 h. (**C**) CCK-8 assay showing significantly higher absorbance in si-*FRZB* compared with si-NC cells. Scale bar = 400 μm. (**D**) Representative EdU staining images of si-NC and si-*FRZB* cells. EdU+ cells (red) indicate S-phase nuclei; nuclei were counterstained with DAPI (blue). Scale bar = 400 μm. (**E**) Relative mRNA expression levels of proliferation markers (*Ki67*, *CDK4*, and *cyclin B*) in si-NC and si-*FRZB* groups measured via qPCR 24 h after transfection. Data are presented as the mean ± SD. ** *p* < 0.01; *** *p* < 0.001.

Skeletal muscle development relies on coordinated processes including myoblast proliferation, migration, differentiation, and myotube fusion. We investigated the role of *FRZB* via RNA interference in C2C12 myoblasts and confirmed that siRNA-1127 achieved the most efficient knockdown of *FRZB* expression (Figure 2A). Functional assays demonstrated that *FRZB* silencing markedly enhanced cell proliferation. Specifically, microscopic observation showed increased cell density (Figure 2B), and the CCK-8 assay revealed significantly higher absorbance values in the si-*FRZB* group than in the si-NC group at 48 h (Figure 2C). Consistently, the EdU assay showed a significant increase in the percentage of EdU-positive nuclei following *FRZB* knockdown (Figure 2D). Moreover, qPCR analysis revealed that *FRZB* depletion significantly upregulated proliferation-associated marker genes including *Ki67*, cyclin dependent kinase 4 (*CDK4*), and *cyclin B* (Figure 2E). Together, these results demonstrated that *FRZB* knockdown enhances the proliferative capacity of C2C12 myoblasts.

### 3.3. FRZB Knockdown Markedly Increases Transwell Migration of C2C12 Myoblasts

**Figure 3:**
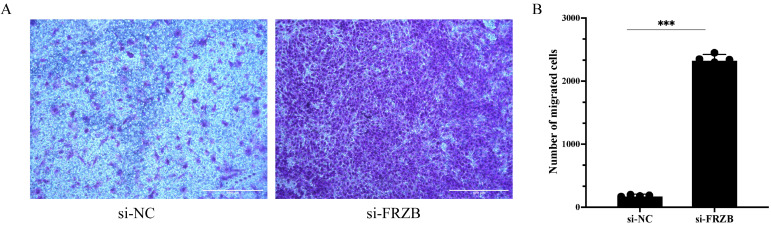
*FRZB* knockdown enhances transwell migration of C2C12 myoblasts. (**A**) Representative crystal violet-stained images showing migrated cells on the lower surface of the transwell membrane in si-NC and si-*FRZB* groups. Scale bar = 400 μm. (**B**) Quantification of migrated cells. For each well, five randomly selected microscopic fields were counted and averaged. Data are presented as the mean ± SD; *** *p* < 0.001 (unpaired two-tailed *t*-test).

To assess whether *FRZB* influences myoblast motility, we performed Transwell migration assays after siRNA-mediated silencing of *FRZB* in C2C12 cells. Crystal violet staining revealed a visibly denser layer of migrated cells on the lower membrane surface in the si-*FRZB* group than that in the si-NC group (Figure 3A). Quantification of the average number of migrating cells in five randomly selected microscopic fields per well confirmed a robust and significant increase in the number of migrated cells following *FRZB* knockdown (*** *p* < 0.001; Figure 3B). These findings indicate that the loss of *FRZB* greatly enhances the migratory capacity of C2C12 myoblasts.

### 3.4. FRZB Knockdown Accelerates Myogenic Differentiation and Augments Myotube Formation in C2C12 Cells

**Figure 4:**
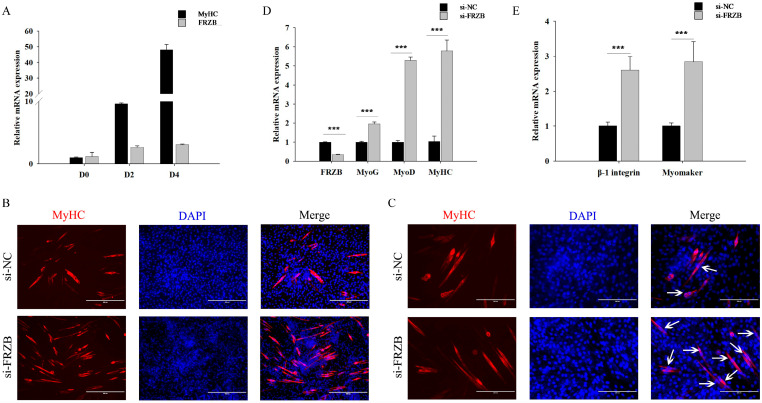
*FRZB* knockdown accelerates myogenic differentiation and enhances myotube fusion in C2C12 cells. (**A**) Time-course qPCR analysis of *MyHC* and *FRZB* mRNA expression at days 0 (D0), 2 (D2), and 4 (D4). (**B**) Representative immunofluorescence staining of *MyHC* (red) after 4 d in differentiation medium, showing the overall increased abundance of myotubes in si-*FRZB* cells; nuclei were counterstained with DAPI (blue). Scale bar = 400 μm. (**C**) Representative high-magnification images highlighting the formation of multinucleated myotubes (white arrows). Scale bar = 200 μm. (**D**) qPCR analysis of myogenic differentiation markers (*MyoG*, *MyoD*, and *MyHC*) at D4. (**E**) qPCR analysis of fusion-related markers (*β1-integrin* and *Myomaker*) at D4. Data are presented as the mean ± SD from three independent experiments. *** *p* < 0.001 (vs. si-NC; Student’s *t*-test).

We first monitored the expression profiles of *MyHC* and *FRZB* during C2C12 differentiation. Time-course qPCR analysis showed that the expression of *MyHC* gradually increased, confirming successful myogenic induction. Similarly, *FRZB* expression also rose throughout the process, reaching its peak on Day 4 (Figure 4A). Subsequent functional assays showed that *FRZB* knockdown markedly accelerated this myogenic progression. Consistently, immunofluorescence (IF) staining after 4 d in differentiation medium revealed an increased overall abundance and size of *MyHC*^+^ myotubes in si-*FRZB* cells (Figure 4B). Higher-magnification imaging further highlighted a significantly higher frequency of multinucleated myotube formation (white arrows, Figure 4C). qPCR on day 4 confirmed significant upregulation of differentiation markers (*MyoG*, *MyoD*, and *MyHC*) (Figure 4D) and fusion-related genes (*β1-integrin* and *Myomaker*) (Figure 4E). Together, these data demonstrate that loss of *FRZB* accelerates myogenic differentiation and enhances myotube growth and fusion.

### 3.5. FRZB Knockdown Skews C2C12 Transcription Toward a Pro-Hypertrophic Profile

**Figure 5:**
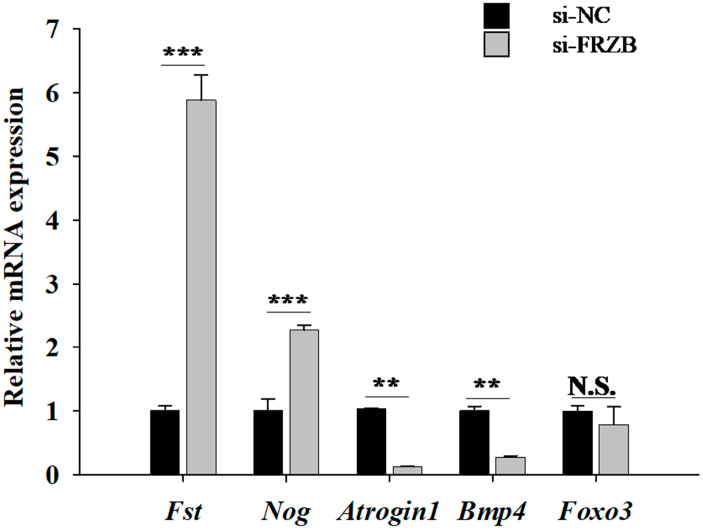
*FRZB* knockdown skews the expression of hypertrophy- and atrophy-associated genes in C2C12 cells. Data are presented as the mean ± SD from three independent experiments. ** *p* < 0.01; *** *p* < 0.001; N.S., no significant (Student’s *t*-test).

To determine whether *FRZB* affects growth-promoting or catabolic programs, we profiled canonical hypertrophy- and atrophy-related genes via qPCR on day 4 of differentiation. *FRZB* knockdown markedly upregulated the hypertrophy-associated genes follistatin (*Fst*) and noggin (*Nog*) and significantly downregulated the atrophy-related E3 ligase *Atrogin1* (Figure 5). In contrast, *Foxo3* showed no significant change (N.S.), and *Bmp4* was also downregulated. Taken together, these findings indicate that *FRZB* depletion shifts the transcriptional landscape toward a pro-hypertrophic and anti-atrophic state, which is consistent with the observed enhancement of myoblast proliferation, differentiation, and myotube formation upon *FRZB* knockdown.

## 4. Discussion

In this study, we showed that *FRZB* is widely expressed across multiple porcine fetal tissues and is relatively elevated in the LD muscle of slow-growing TP/WJ pig breeds compared with the fast-growing LW breed. Although *FRZB* expression was high in the leg muscle and kidney, our focus on the LD stems from its critical role as the primary indicator of meat quantity and quality in porcine production. In vitro, *FRZB* silencing in C2C12 myoblasts promoted their proliferation, migration, differentiation, and myotube fusion while shifting transcriptional programs toward hypertrophy and away from atrophy. Collectively, these results support the notion that *FRZB* acts as a negative regulator of myogenesis.

Mechanistically, *FRZB* (also known as sFRP3) belongs to the secreted frizzled-related protein family and contains a cysteine-rich domain (CRD) homologous to the Wnt-binding region of frizzled receptors, enabling it to bind to Wnt ligands and block their interaction with cell surface receptors [30]. Such binding can attenuate canonical Wnt/β-catenin signaling, which is critical for myogenic progression. Indeed, previous studies have shown that Wnt signaling promotes muscle regeneration and specification. Polesskaya et al. [31] found that Wnt signals can induce myogenic commitment in satellite cells, analogous to fetal myogenesis. In addition, during head muscle development, the Wnt antagonist Frzb collaborates with BMP inhibitors (e.g., noggin) to regulate myogenesis [32]. Thus, our data showing that *FRZB* knockdown enhances myogenic outcomes are consistent with the idea that reducing Wnt antagonism permits more robust activation of downstream myogenic gene programs. Moreover, the regulatory role of *FRZB* in myogenesis likely extends beyond simple Wnt antagonism through complex crosstalk with other signaling networks. For instance, sFRPs can interact with molecules unrelated to the Wnt cascade, such as acting as proteinase inhibitors to interfere with BMP signaling or serving as negative modulators of *ADAM10*, a key enzyme in Notch pathway activation. In skeletal muscle regeneration, Wnt and Notch signaling often act in opposition to control the balance between progenitor proliferation and terminal differentiation [33,34]. Thus, the observed myogenic effects of *FRZB* may represent the integrated output of a multi-layered signaling hub.

Beyond muscle regulation, canonical Wnt/β-catenin signaling is known to inhibit adipogenic differentiation by suppressing adipogenic transcription factors (e.g., C/EBPα and PPARγ). In contrast, extracellular Wnt antagonists may shift the cell fate toward adipogenesis under certain conditions [20,21,35]. While we did not directly test adipogenic markers in our experiment, the relatively higher *FRZB* expression in LD and BF of TP/WJ pigs suggests that elevated *FRZB* in these breeds partly suppresses myogenesis and favors adipose differentiation, contributing to their slower growth and distinct carcass traits. This hypothesis aligns with the established observation that Wnt antagonism generally promotes adipogenesis [22,35].

Placing our findings within a broader signaling landscape, recent reviews have highlighted the complex crosstalk between Wnt/β-catenin and other pathways (Notch, TGF-β, Hippo, and mechanotransduction) in tissue development and homeostasis [24]. Sharma et al. [36] demonstrated that chromatin-remodeling complexes (mSWI/SNF) regulate the accessibility of Wnt pathway genes during myogenic differentiation, indicating a deeper regulatory layer controlling Wnt responsiveness in muscle cells. Furthermore, recent systematic reviews have emphasized the stage-specific roles of Wnt signaling in muscle-bone crosstalk, suggesting that the precise timing of Wnt antagonism is crucial for balanced tissue growth [23]. These studies underscore that the effects of *FRZB* might be modulated by broader epigenetic and signaling networks, potentially explaining why a ubiquitously expressed protein can exert tissue-specific effects on muscle mass.

Interestingly, in the context of neuromuscular diseases, Kwan et al. [37] reported *FRZB* upregulation in limb muscles from patients with amyotrophic lateral sclerosis and mouse models, with expression localized to connective tissue surrounding atrophic fibers. This suggests that *FRZB* is induced by muscle damage or denervation and may inhibit regeneration via Wnt antagonism. This raises the intriguing possibility that in pathological states involving muscle atrophy or denervation, *FRZB* upregulation reduces muscle renewal by suppressing Wnt-mediated myogenic signaling. Our finding that *FRZB* knockdown significantly downregulated the atrophy-related gene *Atrogin1* further supports this link between *FRZB* levels and muscle catabolism.

Despite the findings, our study had some limitations and opens future research directions. First, we acknowledge that the in vivo analysis involved a limited number of sows, which may introduce a nesting effect. However, the consistent molecular trends observed across multiple fetuses provided a rationale for the subsequent mechanistic validation. Second, all functional experiments in this study were conducted in C2C12 cells, which are a classic murine myoblast line. While this model was selected for its high experimental stability and reproducibility compared to primary cells, and despite the high conservation of the FRZB protein sequence and Wnt signaling pathway between pigs and mice, species-specific differences may still exist. Therefore, confirming these inhibitory effects in porcine primary myoblasts or through in vivo experiments remains a necessary step for future research. Third, we inferred a mechanism by which *FRZB* competitively binds to Wnt ligands and reduces downstream signaling, however, we did not directly measure β-catenin stabilization, TCF/LEF target activation, or receptor-ligand binding dynamics. Incorporation of reporter assays (e.g., TOP/FOP), immunoprecipitation of Wnt-receptor complexes, or rescue experiments (e.g., Wnt3a addition) would provide stronger support for this proposed mechanism. Furthermore, we must address potential alternative explanations and genetic compensation within the sFRP family. The sFRP family exhibits significant functional redundancy [38,39]. For instance, transcriptomic analysis of *FRZB*-knockout mice in articular cartilage demonstrated a marked compensatory upregulation of *Sfrp1* and *Sfrp2* to maintain the extracellular Wnt inhibitory tone. While our siRNA-mediated knockdown in the C2C12 model produced a robust myogenic phenotype, we cannot rule out the possibility that the system triggers transcriptional adaptation, a molecular response often mediated by mutant mRNA degradation that upregulates related paralogs to buffer against the loss of a single factor [33,40]. Therefore, the role of FRZB should be viewed as part of a highly resilient and redundant regulatory network, rather than an isolated controller. Finally, although we focused on canonical Wnt signaling, *FRZB* and related sFRPs may also modulate non-canonical Wnt pathways (such as Wnt/Ca^2+^ or planar cell polarity routes) or cross-talk with growth pathways such as PI3K/AKT, MAPK, or integrin signaling. Exploring these axes might reveal additional regulatory mechanisms.

In summary, we demonstrate that *FRZB* suppresses myogenesis via extracellular competition with Wnt ligands, thereby shifting muscle cell behavior toward reduced proliferation, fusion, and hypertrophy. Considering the differential expression of *FRZB* among pig breeds and its known genetic variations, this gene represents a candidate locus for further investigations aimed at improving livestock muscle growth and carcass traits.

## 5. Conclusions

In conclusion, our findings identify Porcine *FRZB* (sFRP3) as a negative regulator of myogenesis. In vitro, *FRZB* knockdown alleviated the suppression of Wnt signaling, thereby enhancing C2C12 myoblast proliferation, migration, differentiation, and myotube fusion, while simultaneously shifting transcriptional programs toward hypertrophy and away from atrophy. The significantly higher *FRZB* expression detected in the fetal longissimus dorsi muscle of slow-growing pig breeds (TP and WJ) compared with fast-growing LW pigs suggests that elevated levels of this Wnt antagonist may impose a molecular constraint on muscle development. Collectively, these results provide a mechanistic basis for the breed-specific differences in muscle growth and highlight *FRZB* as a biologically plausible candidate gene for future applications in porcine molecular breeding and carcass trait improvement.

## Data Availability

The data that support the findings of this study are available from the corresponding author upon reasonable request.

## Supplementary Materials

The following supporting information can be downloaded at: https://www.mdpi.com/article/10.3390/ani16020276/s1, Table S1: Composition and nutrient levels of the experimental diets for pregnant sows (DM basis); Table S2: List of primers and the sequences.

## Abbreviations

The following abbreviations are used in this manuscript:

ALS	Amyotrophic lateral sclerosis
BF	Back fat
Bmp4	Bone morphogenetic protein 4
CCK-8	Cell counting kit-8
CDK4	Cyclin dependent kinase 4
CRD	Cysteine-rich domain
DMEM	Dulbecco’s modified Eagle medium
DSP	Diannan small-eared pig
EdU	5-Ethynyl-2-deoxyuridine
FBS	Fetal bovine serum
Foxo3	Forkhead box o3
FRZB	Frizzled-related protein
Fst	Follistatin
IF	Immunofluorescence
LD	Longissimus dorsi
LL	Landrace pig
LW	Large White pig
MRFs	Myogenic regulatory factors
MyHC	Myosin heavy chain
MyoD	Myogenic differentiation antigen
MyoG	Myogenin
Nog	Noggin
PFA	Paraformaldehyde
qRT-PCR	quantitative real-time PCR
SD	Standard deviation
sFRP3	secreted frizzled-related protein 3
sqRT-PCR	semi-quantitative reverse transcription PCR
TP	Tibetan pig
WJ	Wujin pig
YY	Yorkshire pig
