# Supplementary material for: Porcine FRZB (sFRP3) Negatively Regulates Myogenesis via the Wnt Signaling Pathway
Source: Animals (Basel). 2026 Jan 16;16(2):276. doi: 10.3390/ani16020276 (PMC12837581; doi:10.3390/ani16020276)
Supplement: Supplementary file 1 [file animals-16-00276-s001.zip › Supplementary Table S1.pdf]

Table S1. Composition and nutrient levels of the experimental diets for pregnant sows (DM basis).

**Items**	**Content (%)**
**Ingredients**	
Corn	62.00
Soybean meal	14.00
Wheat bran	20.00
Premix[1]	4.00
**Total**	**100.00**
**Nutrient levels**[2]	
Digestible Energy (MJ/kg)	12.85
Crude Protein (CP)	13.50
Crude Fiber (CF)	6.50
Calcium (Ca)	0.85
Total Phosphorus (P)	0.65

Note: [1]The premix provided the following per kg of diet: Vit A 10,000 IU, Vit D_3 2,000 IU, Vit E 40 IU, Cu 15 mg, Fe 80 mg, Zn 80 mg, Mn 30 mg, I 0.3 mg, Se 0.3 mg. [2]Nutrient levels were calculated values based on the Chinese Feeding Standard for Swine (NY/T 65-2004).
