# Supplementary material for: Porcine FRZB (sFRP3) Negatively Regulates Myogenesis via the Wnt Signaling Pathway
Source: Animals (Basel). 2026 Jan 16;16(2):276. doi: 10.3390/ani16020276 (PMC12837581; doi:10.3390/ani16020276)
Supplement: Supplementary file 1 [file animals-16-00276-s001.zip › Supplementary Table S2.pdf]

Table S2. List of primers and the sequences.

Gene name	Accession number	Primer sequence
β-actin-mouse	NM_007393.5	ATCTGGCACCACACCTTCTACA<br>AAGGTCTCAAACATGATCTGGGT
β-actin-pig	NM_001444420.1	GCCAACCGTGAGAAGATGACT<br>GTGACCCCATCCCCAGAGT
Ki67-mouse	NM_001081117.2	ATCATTGACCGCTCCTTTAGGT<br>GCTCGCCTTGATGGTTCCT
Cyclin B-mouse	NM_172301.3	AATACCTACAGGGTCGTGAAGTGA<br>GCTGTATCATCTTCTTGGGCAC
CDK4-mouse	NM_009870.4	GCTGCTACTGGAAATGCTGACC<br>AGCCTTGGGGGGAAACAGA
MyHC-mouse	NM_010855.3	CAAGTCATCGGTGTTTGTGG<br>TGTCGTACTTGGGCGGGTTC
MyoG-mouse	NM_031189.2	CCATCCAGTACATTGAGCGCCTACA<br>ACGATGGACGTAAGGGAGTGCAGAT
MyoD-mouse	NM_010866.2	CGAGCACTACAGTTGGCGACTAAGAT<br>GCTCCACTATGCTGGACAGGCAGT
β-1 integrin-mouse	NM_010578.2	TTACAAGAGTGCCGTGACAACTG<br>GACTAAGATGCTGCTGCTGTGAG
Myomaker-mouse	NM_025376.3	ATCGCTACCAAGAGGCGTT<br>CACAGCACAGACAAACCAGG
Fst-mouse	NM_001301373.2	TCTCTGCGATGAGCTGTGTC<br>CCTCCTCTTCCTCCGTTTCT
Nog-mouse	NM_008711.2	TGTGGTCACAGACCTTCTGC<br>GTGAGGTGCACAGACTTGGA
Atrogin1-mouse	NM_026346.3	GCAGCTGGATTGGAAGAAGA<br>GAGCAGCTCTCTGGGTTGTT
Bmp4-mouse	NM_001316360.1	CCGGATTACATGAGGGATCT<br>CCTGGGATGTTCTCCAGATG
Foxo3-mouse	NM_001376967.1	ACAAACGGCTCACTTTGTCC<br>CTGTGCAGGGACAGGTTGT
FRZB-mouse	NM_011356.4	TCTGTCCTCCACTTACTGTCAATG<br>GCATCAGTTTTACCCAGTCCAA
FRZB-pig	NM_001243330.1	GGTGGAAGGTTCTATTGCTGAG<br>CGTGCTTGCCGAGGGTT
